# Integrated In Silico Discovery of Thymoquinone Analogs Targeting the Keap1–Nrf2 Pathway for Amyotrophic Lateral Sclerosis Therapy

**DOI:** 10.1002/open.70286

**Published:** 2026-08-18

**Authors:** Jabir C. Nalicho, Petro E. Mabeyo, Andrew S. Paluch, Lucas Paul

**Affiliations:** ^1^ Department of Chemistry Dar es Salaam University College of Education Dar es Salaam Tanzania; ^2^ Department of Chemical Paper and Biomedical Engineering Miami University Oxford Ohio USA

**Keywords:** amyotrophic lateral sclerosis, Keap1–Nrf2 signaling, medicinal chemistry, molecular dynamics, molecular modeling, oxidative stress, QSAR modeling

## Abstract

Oxidative stress drives neuronal vulnerability in amyotrophic lateral sclerosis (ALS), making the Keap1–Nrf2 pathway a vital therapeutic target. While thymoquinone (TQ) modulates this axis, its efficacy is limited by low potency and poor drug‐likeness. We utilized an integrated in silico workflow—including validated QSAR modeling (*R* ^2^ = 0.68, *Q* ^2^_ext_ = 0.66), ADMET profiling, docking, 200 ns molecular dynamics, and MM–PBSA analysis—to identify improved TQ‐derived Keap1 inhibitors. Screening 64 analogs prioritized three leads (CHEMBL3416163, CHEMBL4636830, and CHEMBL221598) with favorable safety and blood–brain barrier permeability. Docking and dynamics confirmed these analogs form stable interactions with Kelch domain hotspots. MM–PBSA calculations revealed significantly enhanced binding free energies (−75.10 to −93.79 kJ mol^−1^) compared to parent TQ (−21.05 kJ mol^−1^), driven primarily by van der Waals and hydrophobic forces. This study identifies structurally tractable TQ analogs with improved predicted potency and establishes a robust computational framework for neuroprotective discovery. The prioritized leads are compelling candidates for in vitro and in vivo validation as redox‐modulating agents in ALS.

## Graphical abstract

Integrated in silico discovery of thymoquinone analogs as potent Keap1 inhibitors for ALS therapy. Using validated QSAR, docking, and 200 ns MD simulations, we identified brain‐penetrant leads (CHEMBL3416163, CHEMBL4636830, and CHEMBL221598) with binding affinities up to fourfold stronger than thymoquinone, offering a targeted strategy to restore redox homeostasis.

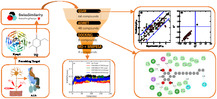

## 1 Introduction

**FIGURE 1:**
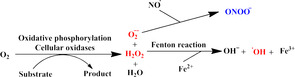
Biochemical pathways leading to the formation of ROS (red) and RNS (blue).

Reactive species are inevitable by‐products of metabolic and environmental processes that continually challenge cellular macromolecules. They comprise both free radicals and nonradical intermediates capable of generating radicals under physiological conditions [1, 2]. Free radicals possess unpaired electrons, which make them highly unstable and prone to abstract electrons from nearby lipids, proteins, and nucleic acids, thereby disrupting cellular integrity and contributing to disease pathologies [2]. At low or regulated concentrations, these species play essential roles in signaling, immune defense, and redox homeostasis [3, 4]. However, when their production exceeds the capacity of antioxidant defenses, it damages vital biomolecules and ultimately drives oxidative stress [5]. Figure 1 illustrates the principal pathways of reactive oxygen and nitrogen species formation that contribute to cellular redox imbalance.

Oxidative stress is implicated across chronic pathologies, including Alzheimer’s and Parkinson’s disease, cancer, cardiovascular disease, diabetes, and amyotrophic lateral sclerosis (ALS) [5, 6]. ALS is a progressive neurodegenerative disorder marked by motor neuron loss, resulting in muscle weakness, paralysis, and typically death from respiratory failure within 3–5 years of symptom onset [7, 8]. Although its etiology is multifactorial, convergent evidence places oxidative stress among the principal drivers of neuronal injury and disease progression [9]. The global burden is substantial and projected to rise with population aging [10, 11]; underrecognition in low‐ and middle‐income settings further underscores the need for effective, brain‐accessible therapies [12, 13].

Approved ALS treatments offer only modest clinical benefit and do not substantially alter long‐term disease progression. Riluzole yields a small survival gain [9], tofersen benefits a genetically defined subgroup [14], and edaravone, while antioxidant‐oriented, shows limited impact on long‐term progression and carries tolerability concerns, including renal toxicity and hypersensitivity reactions [15, 16]. These limitations stem largely from the “antioxidant paradox,” where direct scavengers often fail to match the kinetic scale of reactive species production in the central nervous system. Collectively, this reinforces the rationale for strategies that restore redox homeostasis by re‐engaging endogenous cytoprotective pathways, offering a catalytic antioxidant response, rather than relying solely on direct radical scavenging [17, 18].

**FIGURE 2:**
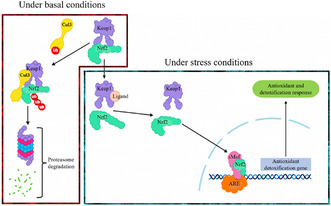
Pharmacological activation of the Keap1–Nrf2 pathway enables transcription of antioxidant and detoxification genes via the antioxidant response element (ARE).

The Keap1–Nrf2 pathway is a master regulator of cellular redox balance [19]. Under basal conditions, Keap1 targets Nrf2 for degradation; during stress, disrupting the Keap1–Nrf2 protein–protein interaction allows Nrf2 to accumulate, translocate to the nucleus, and activate antioxidant and detoxification genes via the antioxidant response element [20]. Importantly, oxidative stress and mitochondrial dysfunction are convergent pathogenic mechanisms across the major genetic forms of ALS, including those associated with *SOD1*, *C9orf72*, *TARDBP*, and *FUS*. Emerging evidence indicates that dysregulation of the Keap1–Nrf2 pathway contributes to this oxidative imbalance, whereas genetic or pharmacological activation of Nrf2 restores redox homeostasis, suppresses mitochondrial oxidative stress, and improves disease‐related phenotypes in experimental ALS models, particularly those associated with *C9orf72* [21, 22]. Directly blocking Keap1 provides an upstream, structurally tractable entry point to strengthen intrinsic defenses before irreversible damage develops [17, 19]. Figure 2 outlines the pharmacological activation sequence of the Keap1–Nrf2 pathway.

**FIGURE 3:**
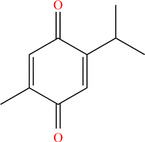
Chemical structure of thymoquinone (TQ, 2‐isopropyl‐5‐methylbenzo‐1,4‐quinone).

Natural products such as flavonoids, sulforaphane, and curcumin provide proof‐of‐concept for Nrf2 activation but commonly suffer from modest potency, off‐target electrophilicity, metabolic instability, and poor blood–brain barrier (BBB) penetration [19, 20, 23, 24]. Thymoquinone (TQ), the principal quinone of *Nigella sativa*, has attracted attention for its antioxidant and neuroprotective effects and can activate Nrf2 by interfering with Keap1 binding (Figure 3) [25, 26]. Importantly, its quinone scaffold offers a chemically tractable, nonelectrophilic framework amenable to rational modification [27]. However, TQ is constrained by poor solubility, rapid metabolism, and low oral bioavailability, necessitating the discovery of analogs that retain the pharmacophoric core while enhancing drug‐likeness and neural reach [28, 29, 30].

To bridge these pharmacological gaps while leveraging the neuroprotective core of TQ, we developed a fully integrated in silico workflow designed to prioritize analogs with optimized Keap1 engagement and enhanced neural reach. By exploring the ChEMBL and DrugBank libraries, we ensured that the resulting candidates possess high translational value and structural tractability [31, 32]. This multistep strategy comprised: (i) scaffold‐aware similarity screening to expand chemical space [33, 34, 35]; (ii) quantitative structure–activity relationship (QSAR) modeling with rigorous internal and external validation [36, 37, 38]; (iii) orthogonal drug‐likeness and Absorption, Distribution, Metabolism, Excretion, and Toxicity (ADMET) triage focused on BBB penetration [39, 40, 41, 42, 43]; (iv) structure‐based docking into the Keap1 Kelch pocket [44, 45]; and (v) 200 ns molecular dynamics with MM–PBSA free‐energy analysis to decompose energetic drivers under dynamic conditions [46, 47].

In summary, this study builds on the recognition that restoring redox balance through endogenous defense pathways offers greater therapeutic promise than direct radical scavenging alone. By focusing on TQ analogs as modulators of the Keap1–Nrf2 pathway, we provide a blueprint for accelerating the rational design of neuroprotective agents grounded in antioxidant biology.

## 2 Materials and Methods

### 2.1 Similarity‐Based Identification of Thymoquinone Analogs

We utilized the SwissSimilarity web server [33] to perform a 2D scaffold‐based similarity search, using the SMILES representation of TQ as the query molecule. To maximize the translational value and biological relevance of our findings, we explored two distinct chemical spaces: the ChEMBL library [31] of bioactive compounds and the DrugBank library [32] of clinically approved drugs. The *Scaffold* similarity method was specifically selected to preserve the essential benzoquinone pharmacophore, the core responsible for redox modulation, while permitting rational peripheral diversification around the isopropyl substituent. This similarity search was conducted using the SwissSimilarity web server [33] (accessed October 26, 2025). Following the removal of duplicate entries, we generated a nonredundant dataset exported in SMILES format for subsequent QSAR modeling.

### 2.2 Quantitative Structure–Activity Relationship (QSAR) Modeling

**FIGURE 4:**
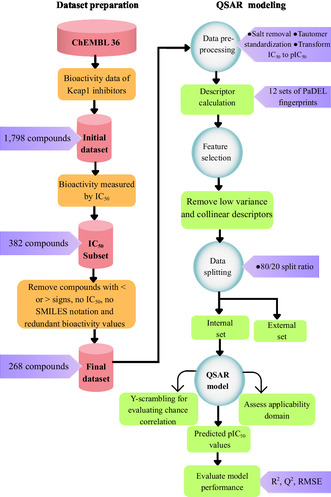
Workflow of the QSAR modeling process applied in this study.

A schematic overview of the QSAR workflow is provided in Figure 4. In summary, we conducted comprehensive QSAR modeling to predict Keap1 inhibition in strict accordance with the guidelines of the Organisation for Economic Co‐operation and Development (OECD) [48]. The modeling framework adhered to four foundational principles: (i) defining a clear biological endpoint; (ii) applying a transparent and reproducible learning algorithm; (iii) establishing a defined applicability domain (AD); and (iv) evaluating performance using robust measures of goodness‐of‐fit and external predictivity.

#### 2.2.1 Data Collection and Preprocessing

Bioactivity data for human Keap1 (ChEMBL target ID: CHEMBL2069156) were retrieved from the ChEMBL database (version 36) [49, 50]. The initial dataset comprised 1798 compounds across 21 assay endpoints. For modeling, we retained only compounds with experimentally determined half‐maximal inhibitory concentration (IC_50_) values in nanomolar units, yielding 382 entries. To ensure data integrity, we removed duplicates (identified by canonical SMILES), records with inequality qualifiers (“<” or “>”), and entries with missing IC_50_ values. The final curated dataset consisted of 268 unique compounds. To normalize activity values for consistent comparison, IC_50_ values were transformed to their negative logarithmic form (pIC_50_) using Equation (1)

(1) $${\mathrm{pIC}}_{50}=-\log_{10}({\mathrm{IC}}_{50}\times 10^{-9})$$

#### 2.2.2 Descriptor Calculation

Prior to computation, all molecular structures were standardized by removing salts, assigning aromaticity, and normalizing tautomeric and nitro groups to ensure chemical consistency [51]. Using PaDEL‐Descriptor, we generated twelve classes of fingerprint‐based molecular descriptors (Table 1) to capture diverse topological, substructural, and electronic characteristics [59].

**TABLE 1:** Summary of twelve fingerprint descriptor sets employed in this study.

Fingerprint	Number	Description	Reference
2D atom pairs	780	Presence of atom pairs at various topological distances	[52]
2D atom pairs count	780	Count of atom pairs at various topological distances	[52]
CDK	1024	Fingerprint of length 1024 and search depth 8	[53]
CDK extended	1024	Extends CDK with additional ring‐feature bits	[53]
CDK graph only	1024	Considers atomic connectivity without bond order	[53]
E‐state	79	Electrotopological state atom types	[54]
Klekota–Roth	4860	Presence of chemical substructures	[55]
Klekota–Roth count	4860	Count of chemical substructures	[55]
MACCS	166	Binary representation of predefined chemical keys	[56]
PubChem	881	Binary representation of PubChem‐defined substructures	[57]
Substructure	307	Presence of SMARTS‐encoded functional groups	[58]
Substructure count	307	Count of SMARTS‐encoded functional groups	[58]

#### 2.2.3 Data Preprocessing and Feature Selection

Descriptor matrices were mean‐centered and scaled to unit variance. To minimize multicollinearity, we removed redundant descriptors with pairwise correlations greater than 0.95 or those with minimal variance (<0.01) [59].

We employed a two‐stage hybrid feature selection protocol to identify an optimal subset of informative descriptors. In the first stage, a Random Forest model was used to rank descriptors by importance, retaining a reduced subset of the most informative features to preserve nonlinear relationships. In the second stage, recursive feature elimination with 10‐fold cross‐validation (RFECV), using a Random Forest base estimator, was applied to iteratively remove weakly informative variables. This procedure yielded final descriptor sets ranging from 29 to 50 features across the twelve fingerprints, which were subsequently used for consistent model training and prediction.

#### 2.2.4 Model Development and Assessment

The dataset was divided into 80% training and 20% external test subsets using stratified random sampling to ensure a balanced distribution of activity values [60]. We selected the Random Forest (RF) algorithm for its ability to capture complex nonlinear relationships and its resistance to overfitting in moderate‐sized datasets [61, 62, 63]. Model implementation used the scikit‐learn library [64] with systematically tuned hyperparameters.

Predictive performance was examined through 10‐fold cross‐validation, external validation (*n *= 54), and Y‐randomization (100 iterations) to ensure the observed correlations were not due to chance. Statistically acceptable models required $R^{2}\geq 0.6$, $Q^{2}\geq 0.5$, and an $R^{2}-Q^{2}$ difference ≤0.2 [65, 66].

#### 2.2.5 Applicability Domain

The applicability domain (AD) defines the chemical space within which a QSAR model is expected to make reliable predictions, as compounds lying outside this region are more likely to yield uncertain outcomes. In this study, we assessed the AD using a Williams plot, which integrates leverage values and standardized residuals to identify structurally influential compounds and potential prediction outliers [67, 68].

Leverage values ($h_{i}$) were calculated according to Equation (2)

(2) $$h_{i}=x_{i}^{T}{\left(X^{T}X\right)}^{-1}x_{i}$$

where $x_{i}$ is the descriptor row vector of the *i*th compound, X is the descriptor matrix, and *X* ^T^ is its transpose. The warning leverage threshold ($h^{*}$) was determined using Equation (3)

(3) $$h^{*}=\frac{3(p+1)}{n}$$

where p is the number of model descriptors and n is the number of training compounds.

Compounds with leverage values exceeding $h^{*}$ were considered structurally influential, whereas those with standardized residuals beyond ±3 standard deviations were classified as outliers. The Williams plot thus provides a visual and statistical means of defining the model’s AD, ensuring that only structurally relevant and statistically reliable predictions are interpreted within the model’s chemical space [37, 68].

### 2.3 Drug‐Likeness and ADMET Properties

We evaluated drug‐likeness and ADMET (absorption, distribution, metabolism, excretion, and toxicity) properties to determine the pharmacokinetic suitability and safety of the QSAR‐predicted TQ analogs. Canonical SMILES for the candidates were submitted to an orthogonal suite of predictive servers, including SwissADME [41], pkCSM [42], and ProTox‐3.0 [43].

To ensure oral suitability, we first applied Lipinski’s “Rule of Five” using the following thresholds: molecular weight (MW) ≤500 Da, hydrogen bond donors (HBD) ≤5, hydrogen bond acceptors (HBA) ≤10, and partition coefficient (Log *P*) ≤5 [39]. We further refined this selection using Veber’s criteria, requiring rotatable bonds (RB) ≤10 and a topological polar surface area (TPSA) ≤140 Å^2^  [40]. Compounds were prioritized if they demonstrated a bioavailability score (BAS) ≥0.55, indicating acceptable oral absorption [69], and a synthetic accessibility (SA) score <5, ensuring they remain structurally tractable for future development [70].

Comprehensive ADME profiling focused on gastrointestinal absorption, P‐glycoprotein (P‐gp) substrate status, and blood–brain barrier (BBB) permeability—a prerequisite for ALS‐directed therapies. We also utilized pkCSM to predict cytochrome P450 interactions and total systemic clearance to assess potential metabolic liabilities. Safety was rigorously assessed across five primary toxicological endpoints: hepatotoxicity, neurotoxicity, nephrotoxicity, carcinogenicity, and mutagenicity. Following a conservative triage strategy, we excluded any compound predicted to be toxic in a single endpoint or classified within Predicted Toxicity Class (PTC) I–III (LD_50_ < 300 mg kg^−1^) [43].

### 2.4 Molecular Docking

We conducted structure‐based molecular docking to evaluate the binding modes and relative affinities of TQ and its prioritized analogs within the Keap1 Kelch domain. TQ and the lead candidates identified through QSAR and ADMET screening were retrieved from PubChem in SDF format [71]. To ensure optimal starting geometries, we performed energy minimization using the MMFF94 force field in Open Babel 3.1.1 [72]. Ligands were subsequently converted to PDBQT format, incorporating polar hydrogens and Gasteiger charges for compatibility with the docking algorithm [72].

The high‐resolution crystal structure of the human Keap1 Kelch domain (PDB ID: 4XMB) was obtained from the Protein Data Bank (PDB) [73]. Receptor preparation was performed using AutoDockTools 1.5.6, which involved removing crystallographic water molecules, adding polar hydrogens, and assigning Kollman charges before exporting the receptor as a PDBQT file [74].

Docking simulations were executed using AutoDock Vina version 1.2.0 [45, 75]. The search grid was centered at the centroid of the native ligand‐binding pocket (x=4.266Å,y=0.692Å,z=−23.196Å) with dimensions of 40Å×40Å×40Å to ensure comprehensive coverage of the interaction region. We employed an exhaustiveness of 8 to generate nine poses per ligand, retaining the conformation with the most negative predicted binding affinity for further analysis [75].

To ensure the reliability of the docking protocol, we performed a self‐docking validation by redocking the cocrystallized ligand into the receptor site [76]. The predicted pose was superimposed onto the experimentally resolved conformation to verify the reproduction of the native binding mode [76]. All ligand–receptor interactions were visualized and analyzed using UCSF Chimera and BIOVIA Discovery Studio [77, 78].

### 2.5 Molecular Dynamics Simulations

The docked Keap1–ligand complexes served as the initial configurations for molecular dynamics (MD) simulations to evaluate their structural stability under simulated physiological conditions. Protein topologies were prepared in GROMACS 2024.3 [79] using the pdb2gmx module with the OPLS‐AA force field [80] and the TIP4P water model [81]. Ligand structures were extracted from the top‐ranked docking poses, hydrogenated, and assigned Gasteiger charges using UCSF ChimeraX [82]. We performed ligand parameterization via the LigParGen web server to generate OPLS‐AA‐compatible coordinates and topology files [83].

The resulting protein–ligand systems were solvated in a dodecahedral TIP4P water box and neutralized with counterions. Energy minimization was conducted using the steepest‐descent algorithm until the maximum force reached 1000 kJ mol^−1^ nm^−1^ [84].

System equilibration was performed in two restrained phases: an NVT phase at 300 K using the stochastic velocity rescaling (v‐rescale) thermostat [85], followed by an NPT phase at 1 bar using the v‐rescale thermostat and the Berendsen barostat [86]. The solvated systems were electrically neutralized by the addition of six Na^+^ counterions prior to energy minimization. Long‐range electrostatics were treated with the Particle‐mesh Ewald (PME) method [87], and all covalent bonds involving hydrogen atoms were constrained using the LINCS algorithm [88], allowing for a 2 fs timestep.

Production simulations were executed for 200 ns under periodic boundary conditions using the leap‐frog integrator [89], with coordinates recorded at 10 ps intervals. To characterize the conformational stability and local flexibility of the complexes, we analyzed the trajectories for root‐mean‐square deviation (RMSD), root–mean‐square fluctuation (RMSF), radius of gyration (*R*_g_), and solvent‐accessible surface area (SASA).

### 2.6 MM–PBSA Binding Free Energy Calculations

MM–PBSA binding free energy calculations were performed using gmx_MMPBSA [90] interfaced with GROMACS 2024.3 [79], employing the Poisson–Boltzmann model for polar solvation energy estimation. Binding free energies were calculated according to Equation (4)

(4) $$\Delta G_{\text{bind}}=G_{\text{complex}}-\left(G_{\text{protein}}+G_{\text{ligand}}\right)$$

where *G*_complex_, *G*_protein_, and *G*_ligand_ represent the free energies of the protein–ligand complex, isolated protein, and isolated ligand, respectively [91]. The free energy of each species was estimated according to

(5) $$G=E_{\text{MM}}+G_{\text{solv}}-T\Delta S$$

where *E*_MM_ is the molecular mechanics energy, *G*_solv_ is the solvation free energy, and TΔS is the entropic contribution [92]. In the present study, the entropic term was omitted to facilitate the comparative ranking of structurally related ligands, consistent with common MM–PBSA practice [46, 91].

The equilibrated portion of each MD trajectory (180–200 ns) was selected based on RMSD stabilization. Snapshots were extracted every 200 ps after removing periodic boundary conditions, recentering the complexes, and stripping solvent molecules and ions prior to MM–PBSA calculations. Per‐residue energy decomposition was subsequently performed following the approach of Homeyer and Gohlke [91] to identify the residues contributing most significantly to ligand binding within the Keap1 Kelch domain.

## 3 Results and Discussion

### 3.1 Model Performance and Validation

Twelve Random Forest‐based QSAR models were developed using distinct molecular fingerprints to identify the structural determinants required for Keap1 inhibition (Table 2). Among these, the **AtomPairs2DCount** model provided the optimal balance between internal robustness and external predictivity, yielding $R_{\mathrm{train}}^{2}=0.68$, $Q_{\mathrm{CV}}^{2}=0.61$, and $Q_{\mathrm{Ext}}^{2}=0.66$ with a streamlined set of 32 descriptors [59]. These statistical metrics exceed the established reliability thresholds of $R^{2}\geq 0.6$ and $Q^{2}\geq 0.5$ for valid QSAR models [65]. Furthermore, the minimal difference between *R* ^2^ and *Q* ^2^ (Δ ≤ 0.1) confirms that the model is statistically sound and possesses a low risk of overfitting [66].

**TABLE 2:** Performance summary of fingerprint‐based QSAR models developed using Random Forest.

Fingerprint	Training set		10‐fold CV set		External set		$R^{2}-Q_{\mathrm{Ext}}^{2}$	$R^{2}-Q_{\mathrm{CV}}^{2}$
	*R* ^2^	RMSE_Tr_	$Q_{\mathrm{CV}}^{2}$	RMSE_CV_	$Q_{\mathrm{Ext}}^{2}$	RMSE_Ext_		
AtomPairs2D	0.52	0.87	0.48	0.82	0.48	0.79	0.04	0.04
AtomPairs2DCount	0.68	0.71	0.61	0.70	0.66	0.64	0.07	0.02
CDK	0.53	0.86	0.51	0.78	0.59	0.71	0.02	0.06
CDK extended	0.57	0.82	0.51	0.75	0.52	0.77	0.06	0.06
CDK graph only	0.55	0.84	0.48	0.81	0.42	0.84	0.07	0.13
E‐State	0.52	0.87	0.43	0.86	0.56	0.73	0.09	0.04
Klekota–Roth	0.58	0.81	0.52	0.77	0.56	0.73	0.06	0.02
Klekota–Roth count	0.53	0.86	0.59	0.73	0.61	0.69	0.05	0.08
MACCS	0.60	0.79	0.49	0.80	0.59	0.71	0.12	0.01
PubChem	0.62	0.78	0.53	0.76	0.64	0.66	0.09	0.03
Substructure	0.60	0.79	0.36	0.90	0.38	0.87	0.24	0.22
Substructure count	0.61	0.79	0.58	0.73	0.59	0.70	0.02	0.02

**FIGURE 5:**
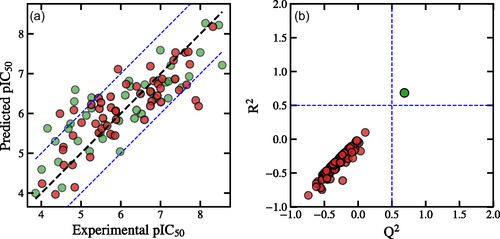
(a) Experimental versus predicted pIC_50_ values for the internal (green) and external (red) sets of the AtomPairs2DCount QSAR model. (b) Y‐scrambling validation showing the actual model (green) versus scrambled models (red). Dashed lines indicate the ideal regression line (black) and acceptance bounds and performance thresholds (blue).

The **SubstructureCount** model also demonstrated acceptable predictive power with an $R_{\mathrm{train}}^{2}$ of 0.61 and a $Q_{\mathrm{Ext}}^{2}$ of 0.59, suggesting its potential utility for consensus‐based modeling frameworks. Assessment of the experimental versus predicted pIC_50_ values for the AtomPairs2DCount model showed that most compounds for both the internal and external sets remained within a ±1.0 error window of the ideal regression line (Figure 5a). This alignment illustrates the model’s capacity to generalize its predictive accuracy across diverse chemical scaffolds.

To ensure the results were not artifacts of random association, Y‐randomization was performed across 100 iterations. The resulting scrambled models produced markedly lower $R^{2}$ and $Q^{2}$ values (<0.2), whereas the actual AtomPairs2DCount model maintained its superior performance, confirming the statistical significance of the observed structure–activity correlations (Figure 5b).

### 3.2 Applicability Domain Analysis

**FIGURE 6:**
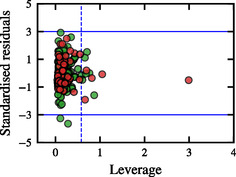
Williams plot illustrating the AD of the QSAR model built using AtomPairs2DCount fingerprints. The plot shows standardized residuals versus leverage values for internal (green) and external (red) sets. Blue horizontal lines represent the ±3 residual boundaries, while the blue dashed vertical line indicates the warning leverage threshold ($h^{*}=0.579$).

We evaluated the applicability domain (AD) of the AtomPairs2DCount model using a Williams plot to ensure that the structural space of the training data was representative of the target chemical space (Figure 6) [68]. A warning leverage threshold ($h^{*}=0.579$) and a standardized residual range of ±3 were established as the definitive cut‐off criteria for reliability [37].

Analysis of the training set identified seven compounds (e.g., CHEMBL4436743, CHEMBL4747827) that exceeded the leverage threshold, while two (e.g., CHEMBL4794829) were identified as residual outliers; notably, no compounds were found to be simultaneously influential and outlying [68]. In the external set, five compounds were identified as structurally influential, but no residual outliers were detected.

The majority of compounds in both the training and test sets resided within the defined chemical space, confirming that the structure–activity relationships are statistically robust and chemically meaningful. This rigorous AD definition ensures that subsequent predictions for TQ analogs remain within the model’s learning domain, thereby strengthening the reliability of the identified leads.

### 3.3 QSAR‐Based Activity Prediction of TQ Analogs

**FIGURE 7:**
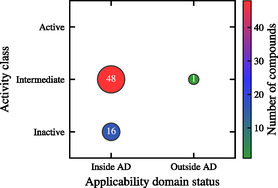
Bubble plot summarizing AD status and predicted activity classes for TQ analogs.

The validated QSAR model built using AtomPairs2DCount fingerprints was applied to predict the inhibitory activity (pIC_50_) of TQ (CHEMBL1672002) alongside 64 structurally related TQ analogs retrieved via SwissSimilarity [33, 34, 35]. Predicted activities were categorized as active (pIC_50_ ≥ 6), intermediate (5 ≤ pIC_50_ < 6), or inactive (pIC_50_ < 5) [93]. The screening identified 49 intermediate and 16 inactive compounds, with no candidates reaching the active threshold (Figure 7).

Applicability domain analysis confirmed that 64 of the compounds resided within the model’s chemical space, while one fell outside, emphasizing the necessity of domain validation for reliable predictions [65, 68]. From this pool, 48 intermediate compounds were prioritized for further analysis. Although TQ was predicted to be inactive (pIC_50_ = 4.01), it remained within the AD and was retained as the reference scaffold for all subsequent comparisons.

The predicted activities and key drug‐likeness descriptors for the top‐performing candidates are summarized in Table 3, while full results for the entire library are provided as a separate CSV file in the Supplementary Material. This methodology follows established integrative frameworks that combine QSAR potency prediction with pharmacokinetic profiling to identify Keap1–Nrf2 inhibitors [94].

**TABLE 3:** Predicted activity, drug‐likeness (Lipinski), and oral bioavailability (Veber) parameters for the shortlisted TQ analogs.

ID	Predicted pIC_50_	Lipinski’s rule of five				Veber’s rule		BAS	SA
		MW	HBA	HBD	Log *P*	RB	TPSA		
CHEMBL4636830	5.50	330.46	3	1	4.79	9	54.37	0.85	4.04
CHEMBL3416352	5.48	304.38	4	0	3.24	7	52.60	0.85	3.81
DB08690	5.48	318.41	4	0	3.73	7	52.60	0.85	3.88
CHEMBL3416172	5.47	294.39	4	1	3.63	10	63.60	0.85	3.68
CHEMBL221137	5.47	294.39	4	2	3.68	10	74.60	0.85	3.66
CHEMBL4096012	5.46	296.36	5	1	2.29	10	72.83	0.85	3.58
CHEMBL221598	5.45	252.31	4	2	2.60	7	74.60	0.85	3.33
CHEMBL3416171	5.45	266.33	4	1	2.88	8	63.60	0.85	3.46
CHEMBL3416180	5.45	280.36	4	0	3.13	9	52.60	0.85	3.55
CHEMBL3416163	5.45	280.36	4	2	3.34	9	74.60	0.85	3.55
CHEMBL1672002 (TQ)	4.01	164.20	2	0	1.85	1	34.14	0.55	2.83

Abbreviations: BAS, bioavailability score; HBA, hydrogen bond acceptors; HBD, hydrogen bond donors; Log *P*, partition coefficient; MW, molecular weight; RB, rotatable bonds; SA, synthetic accessibility; TPSA, topological polar surface area.

### 3.4 Drug‐Likeness and Oral Bioavailability Profiling

Physicochemical screening of the 48 prioritized analogs using Lipinski’s and Veber’s criteria identified ten compounds, along with TQ, that met all thresholds for drug‐likeness (Table 3) [39, 40]. Their molecular parameters, including molecular weight (MW), partition coefficient (log *P*), hydrogen‐bond donors (HBD) and acceptors (HBA), and polar surface area (TPSA), fell within established ranges for favorable permeability and absorption [41]. Notably, the selected analogs exhibited higher predicted activity than TQ, suggesting that scaffold optimization enhances both potency and pharmacokinetic potential, which is consistent with previous reports that structural modification can improve the biological activity of the TQ core [27].

Molecular weights for the shortlisted candidates ranged from 164.20 to 330.46 Da, well within the optimal window (≤500 Da) for passive membrane diffusion [39]. Calculated log *P* values (1.85–4.79) reflected a balanced hydrophilic–lipophilic profile conducive to both aqueous solubility and membrane permeability. Hydrogen‐bond donors (0–2) and acceptors (2–5) were significantly below the recommended limits of 5 and 10, respectively, which favors efficient desolvation and target binding. Furthermore, the number of rotatable bonds (1–10) and the TPSA values (34.14–74.60 Å^2^) satisfied Veber’s thresholds for high intestinal absorption and oral bioavailability [40].

Most analogs achieved a bioavailability score (BAS) of 0.85, compared to 0.55 for TQ, indicating superior oral absorption potential [69]. Synthetic accessibility (SA) scores ranged from 2.83 to 4.04, suggesting that these compounds possess moderate synthetic complexity and are amenable to further chemical optimization [70]. Because all eleven compounds complied with Lipinski’s and Veber’s rules, they were advanced to toxicological evaluation to establish their safety profiles.

### 3.5 Toxicological Evaluation

Toxicity profiling of the eleven drug‐like compounds identified by Lipinski’s and Veber’s filters was performed across several endpoints, including hepatotoxicity (*dili*), neurotoxicity (*neuro*), nephrotoxicity (*nephro*), carcinogenicity (*carcino*), mutagenicity (*mutagen*), and predicted toxicity class (PTC) [43]. Three analogs (CHEMBL3416172, CHEMBL4096012, and CHEMBL3416171) exhibited predicted toxicity in at least one category—specifically nephrotoxicity—and were subsequently excluded from the pipeline [43].

The remaining eight analogs, along with TQ, were predicted to be inactive across all evaluated endpoints and were classified within PTC classes 4–5, indicating low acute toxicity (LD_50_ > 300 mg kg^−1^) [43]. The complete toxicity profiles are presented in Table 4. This early‐stage safety filtering is a critical step in identifying and eliminating potentially reactive or organ‐specific toxicants before advancing to expensive structural simulations [95].

**TABLE 4:** Predicted toxicity profiles of the eleven drug‐like TQ analogs.

ID	Dili	Neuro	Nephro	Carcino	Mutagen	PTC
CHEMBL4636830	Inactive	Inactive	Inactive	Inactive	Inactive	5
CHEMBL3416352	Inactive	Inactive	Inactive	Inactive	Inactive	5
DB08690	Inactive	Inactive	Inactive	Inactive	Inactive	5
CHEMBL3416172	Inactive	Inactive	Active	Inactive	Inactive	5
CHEMBL221137	Inactive	Inactive	Inactive	Inactive	Inactive	4
CHEMBL4096012	Inactive	Inactive	Active	Inactive	Inactive	5
CHEMBL221598	Inactive	Inactive	Inactive	Inactive	Inactive	4
CHEMBL3416171	Inactive	Inactive	Active	Inactive	Inactive	5
CHEMBL3416180	Inactive	Inactive	Inactive	Inactive	Inactive	5
CHEMBL3416163	Inactive	Inactive	Inactive	Inactive	Inactive	4
CHEMBL1672002 (TQ)	Inactive	Inactive	Inactive	Inactive	Inactive	5

Abbreviations: carcino, carcinogenicity; dili, hepatotoxicity; mutagen, mutagenicity; nephro, nephrotoxicity; neuro, neurotoxicity; PTC, predicted toxicity class.

### 3.6 ADME Profiling

ADME evaluation of the eight nontoxic, drug‐like analogs revealed uniformly favorable pharmacokinetic characteristics (Table 5). All compounds exhibited high predicted gastrointestinal absorption and blood–brain barrier (BBB) permeability, with no P‐glycoprotein efflux liability. The consistent BBB penetration across the subset is particularly significant for ALS therapy, as it ensures that the candidates can reach the target Keap1–Nrf2 proteins within the central nervous system.

**TABLE 5:** Predicted pharmacokinetic properties (ADME) of TQ and its analogs.

ID	Absorption			Distribution	Metabolism			Excretion
	WSC	GIA	P‐gp sub	BBB	CYP3A4 sub	CYP3A4 inh	CYP1A2 inh	Total CL
CHEMBL4636830	Moderate	High	No	Yes	Yes	No	No	1.61
CHEMBL3416352	Soluble	High	No	Yes	Yes	No	No	0.31
DB08690	Moderate	High	No	Yes	Yes	No	No	1.58
CHEMBL221137	Moderate	High	No	Yes	Yes	No	No	1.52
CHEMBL221598	Soluble	High	No	Yes	No	No	No	1.44
CHEMBL3416180	Soluble	High	No	Yes	Yes	No	No	1.66
CHEMBL3416163	Moderate	High	No	Yes	No	No	No	1.50
CHEMBL1672002 (TQ)	Soluble	High	No	Yes	No	No	No	0.23

Abbreviations: BBB, blood–brain barrier permeability; GIA, gastrointestinal absorption; P‐gp sub, P‐glycoprotein substrate; WSC, water solubility class; Total CL, total clearance.

Regarding metabolic stability, none of the analogs showed inhibitory activity toward the major cytochrome P450 isoforms CYP1A2 or CYP3A4, reducing the risk of clinically relevant drug–drug interactions. However, substrate status varied among the candidates. CHEMBL221598 and CHEMBL3416163 emerged as the most favorable leads because they combined CYP3A4 nonsubstrate behavior with suitable predicted pharmacokinetic characteristics, whereas the remaining analogs were predicted to be CYP3A4 substrates with varying metabolic turnover potentials. Despite these metabolic differences, candidate prioritization was based on an integrated assessment of absorption, BBB permeability, CYP interaction profile, predicted clearance, and subsequent target engagement, rather than on any single ADME parameter. Although SwissADME [41] and pkCSM [42] predicted TQ to be soluble, this reflects a structure‐based computational estimate rather than experimentally determined aqueous solubility. Previous experimental studies [28, 29, 30] have consistently demonstrated that TQ exhibits poor aqueous solubility, rapid metabolism, and limited oral bioavailability, all of which contribute to its suboptimal pharmacokinetic performance. Consequently, analogs such as CHEMBL3416163 were prioritized despite their moderate predicted solubility because they achieved a more favorable overall balance between pharmacokinetic properties and predicted target engagement. Although their predicted clearance exceeded that of TQ, this was not considered prohibitive, as they simultaneously exhibited favorable BBB permeability, lack of CYP3A4 substrate liability, and substantially stronger predicted binding to Keap1, collectively supporting their selection for further investigation. This integrated ADME–docking framework enabled the prioritization of candidates with favorable pharmacokinetic characteristics while maintaining strong potential for Keap1 target engagement.

### 3.7 Molecular Docking

#### 3.7.1 Docking Protocol Validation

**FIGURE 8:**
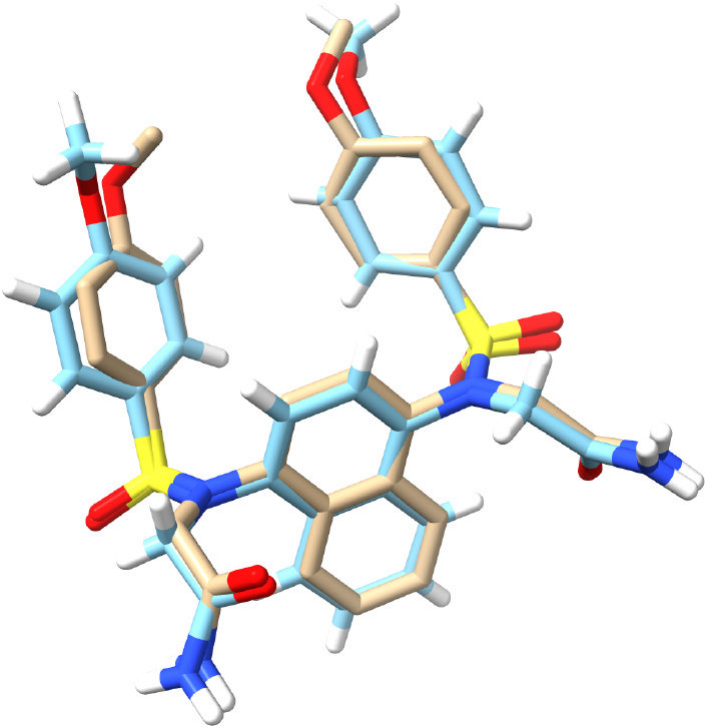
Superposition of the redocked ligand (brown) and the cocrystallised ligand (cyan) within the Keap1 binding pocket.

Prior to the docking experiments, the protocol was validated to ascertain its reliability and accuracy. The cocrystallized ligand was redocked into the Keap1 active site (PDB ID: 4XMB), and the resulting pose was superimposed onto the experimentally resolved conformation, yielding an RMSD of 0.46 Å (Figure 8). This value, being significantly below the generally accepted 2.0 Å threshold, confirms that the docking procedure robustly reproduces the native binding mode and is therefore suitable for subsequent evaluation of the TQ analogs.

#### 3.7.2 Docking Affinity Analysis

Docking simulations against the Keap1 Kelch domain revealed that all eight analyzed analogs exhibited significantly stronger binding affinities than the parent TQ scaffold (−6.1 kcal/mol), as summarized in Table 6. To ensure the selection of leads with the highest translational potential, we integrated these structure‐based results with the previously determined ADME profiles. CHEMBL4636830 was identified as the most potent binder with an affinity of −8.0 kcal/mol. Despite its predicted status as a CYP3A4 substrate, it was prioritized due to this superior binding strength.

**TABLE 6:** Predicted binding affinities of TQ and its analogs against Keap1.

Compound	Affinity, kcal/mol
CHEMBL4636830	−8.0
DB08690	−7.8
CHEMBL3416352	−7.3
CHEMBL3416163	−7.2
CHEMBL221598	−7.2
CHEMBL221137	−7.2
CHEMBL3416180	−6.3
CHEMBL1672002 (TQ)	−6.1

CHEMBL3416163 and CHEMBL221598, both −7.2 kcal/mol, were advanced as high‐priority leads because they combined favorable binding affinities with optimal CYP3A4 nonsubstrate behavior, reducing the risk of metabolic liability. In contrast, the remaining analogs, including CHEMBL3416180, DB08690, CHEMBL221137, and CHEMBL3416352, were deprioritized because their predicted CYP3A4 substrate status outweighed their docking performance. This integrated ADME–docking framework yielded three high‐quality TQ analogs, which, along with the TQ reference, were advanced to dynamic simulation and free‐energy analysis to verify the stability of their binding modes.

#### 3.7.3 Binding Interaction Analysis

**FIGURE 9:**
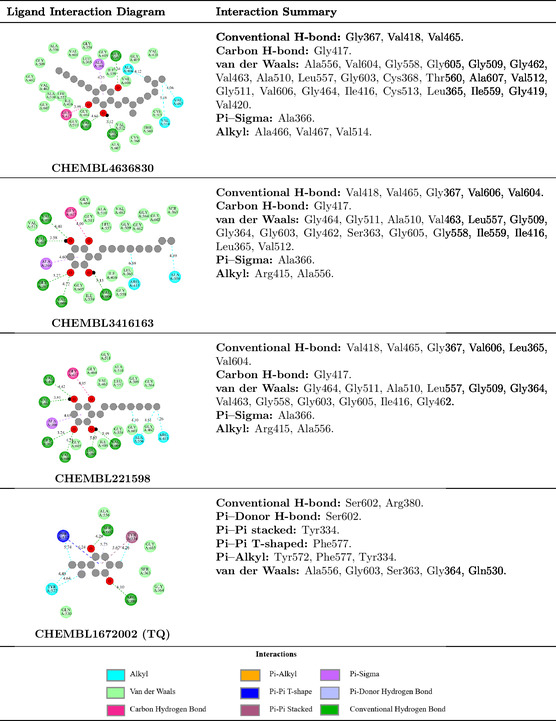
Ligand–Keap1 interaction diagrams and summary of key residue interactions for the four prioritized compounds.

The four prioritized ligands exhibited consistent and residue‐dense binding within the Keap1 Kelch pocket, as illustrated in the interaction panels (Figure 9). CHEMBL4636830, the most potent binder, formed hydrogen bonds with Gly367, Val418, and Val465, reinforced by extensive van der Waals contacts centered on **Gly603**, **Val604**, **and** **Gly511**.

Analogs CHEMBL3416163 and CHEMBL221598 displayed highly consistent binding patterns, characterized by recurrent hydrogen bonds involving Val418, Gly367, and Val606, carbon–hydrogen stabilization at **Gly417**, and a broad van der Waals network spanning **Gly364**, **Gly603**, **and** **Gly511**. In contrast, TQ (CHEMBL1672002) showed a comparatively narrow interaction profile dominated by polar contacts with Ser602 and Arg380, accompanied by limited aromatic interactions such as a single π–π stacking contact with Tyr334. Collectively, the prioritized analogs exploited a broader and more coherent hotspot network, particularly involving Gly364, Val604, and Gly603, providing a structural rationale for their superior affinity compared to the parent scaffold.

### 3.8 Molecular Dynamics Trajectory Analyses

#### 3.8.1 RMSD Analysis

**FIGURE 10:**
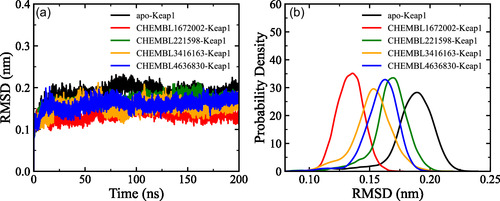
(a) RMSD trajectories of apo‐Keap1 and ligand‐bound complexes over 200 ns. (b) RMSD probability density distributions illustrating the conformational preferences of each system.

RMSD was used to evaluate the structural stability of Keap1 in its apo form and in complex with TQ and its analogs. As shown in Figure 10a, all systems equilibrated rapidly and remained stable over the 200 ns production run, with RMSD values consistently well below the 0.30 nm threshold. Apo‐Keap1 exhibited the highest mean RMSD (0.186 ± 0.018 nm), whereas ligand binding consistently reduced backbone fluctuations, indicating that the candidates enhance the structural rigidity of the Kelch domain. TQ (CHEMBL1672002) produced the lowest mean RMSD (0.134 ± 0.011 nm), while CHEMBL3416163 showed the closest stability profile (0.152 ± 0.016 nm), identifying it as the analog most capable of reproducing the reference scaffold’s stabilizing effect. Although CHEMBL4636830 and CHEMBL221598 stabilized at slightly higher values (0.159–0.167 nm), they maintained structural integrity without evidence of global unfolding or ligand dissociation.

The RMSD probability density distributions (Figure 10b) reinforce these stability trends. While apo‐Keap1 sampled a broader conformational ensemble, the ligand‐bound systems exhibited narrower, left‐shifted unimodal peaks, which are characteristic of restrained backbone motion and restricted conformational space. TQ displayed the most compact distribution (∼0.136 nm), and CHEMBL3416163 exhibited the tightest distribution among the analogs, further confirming its role as a robust stabilizer. These results highlight CHEMBL3416163 as the most promising TQ‐like stabilizer in the set.

#### 3.8.2 RMSF Analysis

**FIGURE 11:**
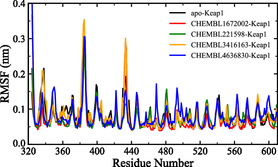
RMSF profiles of apo‐Keap1 and ligand‐bound complexes over 200 ns.

RMSF was used to assess residue‐level flexibility across the Keap1 Kelch domain (residues 327–610) and to determine how TQ and its analogs influence local dynamics (Figure 11). Apo‐Keap1 showed the highest overall fluctuations (mean RMSF = 0.081 nm), with pronounced peaks in the intrinsically mobile loop segments around residues 333–340, 380–388, and 430–437. Ligand binding reduced these motions to varying extents, indicating a general stabilizing effect on the protein structure.

TQ (CHEMBL1672002) exhibited the lowest mean RMSF (0.070 nm) and effectively suppressed mobility in key loops, including 381–388, 397–404, and 429–436. Among the analogs, CHEMBL221598 demonstrated a comparable stabilizing effect (mean RMSF = 0.074 nm), specifically dampening motion across residues 382–388, 397–402, and 478–483. This identifies CHEMBL221598 as the analog most consistent with the parent TQ in reducing local residue flexibility.

In contrast, CHEMBL3416163 and CHEMBL4636830 displayed moderately higher flexibility (0.084 and 0.083 nm, respectively), with elevated peaks at residues 324, 384, and 433. While these peaks occur in regions known to be inherently dynamic and do not indicate global destabilization, they reflect a weaker local stabilization relative to TQ and CHEMBL221598. Overall, the RMSF profiles confirm CHEMBL221598 as the analog that best approximates TQ’s flexibility‐dampening behavior.

#### 3.8.3 SASA Analysis

**FIGURE 12:**
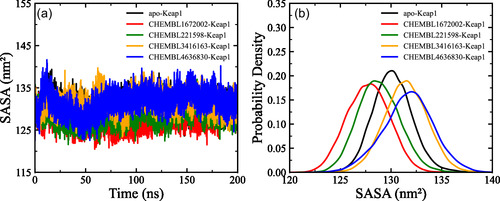
(a) SASA trajectories of apo‐Keap1 and ligand‐bound complexes over 200 ns. (b) SASA probability density distributions illustrating solvent‐exposure preferences for each system.

SASA was analyzed to assess how ligand binding influences the compactness and solvent exposure of the Keap1 Kelch domain (Figure 12a). Apo‐Keap1 exhibited a mean SASA of 130.15 nm^2^, reflecting a higher degree of surface exposure compared to most ligand‐bound systems. TQ (CHEMBL1672002) produced the lowest mean SASA (127.75 ± 2.07 nm^2^), indicating that the reference scaffold promotes a more compact protein conformation with reduced solvent accessibility. Among the analogs, CHEMBL221598 closely approximated this effect with a mean SASA of 128.80 ± 2.05 nm^2^, identifying it as the most effective analog for reproducing TQ‐like structural compaction.

In contrast, CHEMBL3416163 and CHEMBL4636830 displayed higher SASA values (131.47–131.69 nm^2^), which are consistent with modestly expanded surface regions and increased conformational breathing. These trends are further supported by the SASA probability distributions (Figure 12b), where TQ and CHEMBL221598 exhibited sharp, left‐shifted peaks indicative of highly compact states. Conversely, the distributions for CHEMBL3416163 and CHEMBL4636830 were broader and right‐shifted, reflecting increased solvent exposure and higher conformational variability. Collectively, the SASA results reinforce the selection of CHEMBL221598 as the analog that best mirrors the favorable compacting behavior of the parent TQ scaffold.

#### 3.8.4 *R*_g_ Analysis

**FIGURE 13:**
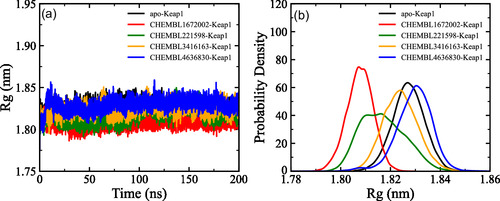
(a) *R*_g_ trajectories of apo‐Keap1 and ligand‐bound complexes over 200 ns. (b) *R*_g_ probability density distributions illustrating global compactness of each system.

The radius of gyration (*R*_g_) was evaluated to assess the global compactness of Keap1 in its apo state and upon ligand binding (Figure 13a). Apo‐Keap1 exhibited a mean *R*_g_ of 1.827 nm, indicating a greater overall expansion compared with the ligand‐bound systems. TQ (CHEMBL1672002) yielded the lowest mean *R*_g_ (1.808 ± 0.005 nm), confirming its ability to promote a more compact and tightly packed protein conformation. Among the candidates, CHEMBL221598 followed with the second‐lowest mean *R*_g_ (1.817 ± 0.009 nm), demonstrating a similarly strong compacting effect.

In contrast, CHEMBL3416163 and CHEMBL4636830 showed modestly elevated *R*_g_ values (1.824–1.830 nm), consistent with slight structural expansion and increased breathing motions of the Kelch domain. These differences were corroborated by the *R*_g_ probability density distributions (Figure 13b), where TQ displayed a narrow, left‐shifted peak characteristic of a highly compact structure, whereas the distributions for CHEMBL3416163 and CHEMBL4636830 were broader and right‐shifted. Overall, the *R*_g_ findings complement the RMSD, RMSF, and SASA trends, identifying CHEMBL221598 as the analog that most closely approximates the compactness induced by the parent TQ scaffold.

### 3.9 MM–PBSA Binding Free Energy Analysis

#### 3.9.1 Total Binding Free Energies

Molecular mechanics Poisson–Boltzmann surface area (MM–PBSA) calculations were employed to quantify the thermodynamic strength of Keap1–ligand interactions, providing a more rigorous validation of the docking‐predicted affinities and the stability profiles obtained from MD simulations. All ligand‐bound systems remained stable over the 200 ns trajectories, providing reliable ensembles for energy estimation. The reference ligand TQ (CHEMBL1672002) exhibited the weakest affinity with a total binding free energy of $\Delta G_{\text{bind}}=-21.05\text{kJ/mol}$, whereas the three prioritized analogs demonstrated markedly stronger binding: CHEMBL3416163 (−93.79 kJ/mol), CHEMBL4636830 (−88.68 kJ/mol), and CHEMBL221598 (−75.10 kJ/mol). This energy ranking mirrors the docking results, confirming that the superior binding strengths of these analogs persist under dynamic sampling conditions.

Analysis of the individual energy components (Table 7) revealed that van der Waals interactions were the dominant stabilizing factors across all complexes, supported by moderate electrostatic contributions. Conversely, polar solvation energies opposed binding in every system, reflecting the significant desolvation penalty required to bury polar functional groups within the predominantly hydrophobic Kelch pocket [96]. The nonpolar solvation term provided modest stabilization, aligning with the structural compaction trends and the expected hydrophobic character of the Kelch‐domain interactions [97]. These energetic drivers are consistent with previous studies of Keap1 inhibitors, where van der Waals forces were identified as the primary determinants of affinity [47, 98].

**TABLE 7:** MM–PBSA energy components (mean ± SD, kJ mol^−1^) for Keap1–ligand complexes.

Ligand complexes	Δ*E*_MM_		Δ*G*_Solv_		Δ*G*_bind_, kJ mol^−1^
	Δ*E*_elec_	Δ*E*_vdW_	Δ*G*_polar_	Δ*G*_non‐polar_	
CHEMBL3416163	−13.870 ± 0.619	−116.484 ± 1.074	51.769 ± 0.811	−15.207 ± 0.125	−93.787 ± 1.150
CHEMBL4636830	−16.520 ± 0.620	−153.961 ± 1.045	102.178 ± 0.838	−20.383 ± 0.097	−88.679 ± 1.431
CHEMBL221598	−26.780 ± 0.676	−126.422 ± 0.862	94.466 ± 0.740	−16.341 ± 0.079	−75.099 ± 1.034
CHEMBL1672002 (TQ)	−2.308 ± 0.462	−24.443 ± 1.938	9.861 ± 3.411	−4.070 ± 0.361	−21.053 ± 3.099

*Note*: Δ*E*_MM_, molecular mechanics energy; Δ*E*_elec_, electrostatic energy; Δ*E*_vdW_, van der Waals energy; Δ*G*_Solv_, solvation free energy; Δ*G*_polar_, polar solvation energy; Δ*G*_non‐polar_, nonpolar solvation energy; Δ*G*_bind_, total binding free energy.

#### 3.9.2 Per‐Residue Binding Energy Contributions

**FIGURE 14:**
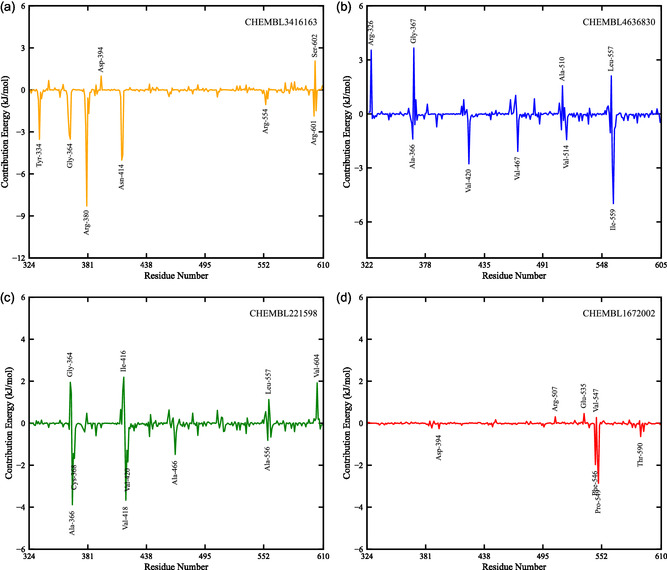
Per‐residue MM–PBSA energy decomposition (kJ mol^−1^) for four Keap1–ligand complexes. Key stabilizing residues are labeled in each panel.

Residue‐level energy decomposition was performed to identify the specific amino acids responsible for stabilizing the complexes within the Keap1 Kelch domain (Figure 14). TQ exhibited only weak interactions, primarily with Pro549 and Phe546, consistent with its limited interaction network and poor binding free energy. In contrast, the prioritized analogs established a much broader and more persistent contact network. CHEMBL3416163 was stabilized most strongly by Arg380 and Asn414, CHEMBL4636830 by Ile559 and Val420, and CHEMBL221598 by Ala366 and Val418. These residues significantly overlap with the structural hotspots identified during docking, confirming that the stabilizing interactions observed in static poses are maintained under dynamic conditions.

Many of the most significant contributors are located within flexible loop regions. The binding of these analogs effectively dampens local fluctuations in these loops, thereby enhancing the global conformational stability of the protein–ligand complex [98]. This stabilization mechanism, combining extensive hydrophobic anchoring with selective polar recognition, provides a coherent structural rationale for why the analogs achieve markedly superior $\Delta G_{\text{bind}}$ values compared to TQ. These findings further support the selection of CHEMBL3416163 and CHEMBL4636830 as the most promising candidates for future lead optimization.

The consistent agreement across QSAR prediction, ADMET profiling, molecular docking, molecular dynamics simulations, and MM–PBSA free‐energy calculations provides convergent computational evidence supporting the prioritization of these TQ analogs as promising Keap1 inhibitors. Nevertheless, these findings remain predictive in nature and should be interpreted within the scope of an in silico investigation. While the integrated computational workflow offers a robust framework for rational lead prioritization, it cannot by itself establish biological activity or therapeutic efficacy. Consequently, experimental studies, including in vitro validation of Keap1–Nrf2 modulation followed by pharmacokinetic and efficacy evaluation in appropriate ALS models, are required to confirm the translational potential of the prioritized analogs.

#### 3.9.3 Biological Implications for ALS and Medicinal Relevance of the Prioritized Analogs

The superior binding stability and favorable binding free energies exhibited by the prioritized analogs are consistent with an enhanced capacity to disrupt the Keap1–Nrf2 protein–protein interaction relative to the parent TQ scaffold. Given the central role of the Keap1–Nrf2 pathway in regulating cellular redox homeostasis, stable occupation of the Kelch binding pocket is expected to promote Nrf2 activation and the expression of cytoprotective genes, a mechanism that has been shown to alleviate oxidative stress, preserve mitochondrial function, and attenuate motor neuron degeneration in experimental ALS models [21, 22, 99].

The identified leads also possess encouraging medicinal relevance. CHEMBL4636830 corresponds to cannabigeroquinone, a member of the cannabinoquinone family whose analogs have progressed into clinical development for inflammatory and neurodegenerative disorders [100]. CHEMBL221598 is structurally related to embelin, a bioactive benzoquinone with established antioxidant and anti‐inflammatory properties that has recently been investigated in experimental ALS research [101]. Meanwhile, CHEMBL3416163 represents a comparatively underexplored alkyl‐substituted 2,5‐dihydroxy‐1,4‐benzoquinone scaffold, a class recognized for its chemical tractability and diverse pharmacological activities [102, 103, 104]. Collectively, the established medicinal relevance of these scaffolds complements the computational findings presented here and further supports their prioritization as promising noncovalent Keap1 inhibitor scaffolds for future investigation.

## 4 Conclusion

This study identifies thymoquinone (TQ) analogs with substantially enhanced Keap1 affinity and drug‐like features through a rigorously integrated computational workflow. By combining a validated QSAR model ($R^{2}=0.68,Q_{\text{ext}}^{2}=0.66$) with ADMET triage, molecular docking, and 200 ns molecular dynamics simulations, three prioritized analogs (CHEMBL3416163, CHEMBL4636830, and CHEMBL221598) emerged as consistently superior to the parent TQ scaffold. These leads exhibited markedly stronger binding free energies, ranging from −75 to  −94 kJ/mol compared to −21.05 kJ/mol for TQ, characterized by broader hotspot engagement within the Kelch pocket and stable dynamic behavior supported by restrained backbone flexibility. Per‐residue energy decomposition confirmed that binding is primarily driven by van der Waals and hydrophobic interactions, providing a structural rationale for the superior potency of these analogs.

Beyond identifying high‐quality candidates, this work establishes a coherent in silico framework for the rational discovery of Keap1 inhibitors relevant to ALS and other redox‐driven neurodegenerative conditions. The prioritized analogs combine improved binding affinity with predicted oral bioavailability and blood–brain barrier permeability, positioning them as credible starting points for experimental validation. Immediate future efforts should focus on confirming Keap1–Nrf2 modulation in vitro, followed by pharmacokinetic and efficacy assessments in ALS models and iterative structure–activity refinement. Overall, these findings demonstrate that rational modification of the TQ scaffold can yield tractable Keap1 inhibitors with significant translational potential for redox‐centered neuroprotection.

## Author Contributions

**Jabir C. Nalicho:** conceptualization, data curation, formal analysis, investigation, methodology, validation, visualization, writing – original draft, writing – review & editing. **Petro E. Mabeyo:** supervision, validation, writing – review & editing. **Andrew S. Paluch:** resources, supervision, software, writing – review & editing. **Lucas Paul:** project administration, resources, supervision, validation, writing – review & editing.

## Conflicts of Interest

The authors declare no conflicts of interest.

## Supporting information

Predicted inhibitory activities (pIC_50_), activity classes, leverage values, and applicability domain (AD) status for the complete library of 64 thymoquinone analogs obtained from SwissSimilarity using the validated AtomPairs2DCount QSAR model are provided as a CSV file on Zenodo at https://doi.org/10.5281/zenodo.18483747. Additional information and details may be made available by contacting the corresponding author.

## Data Availability

The data that support the findings of this study are openly available in Zenodo at https://doi.org/10.5281/zenodo.18483747.
